# Supplementary material for: Machine and deep learning algorithms for sentiment analysis during COVID-19: A vision to create fake news resistant society
Source: PLoS One. 2024 Dec 19;19(12):e0315407. doi: 10.1371/journal.pone.0315407 (PMC11658524; doi:10.1371/journal.pone.0315407)
Supplement: S1 File — (DOCX) [file pone.0315407.s001.docx]

The full code used in this article can be found on the link: <https://github.com/tayyabawan786/covid19-project/tree/main>
