# Supplementary material for: Machine and deep learning algorithms for sentiment analysis during COVID-19: A vision to create fake news resistant society
Source: PLoS One. 2024 Dec 19;19(12):e0315407. doi: 10.1371/journal.pone.0315407 (PMC11658524; doi:10.1371/journal.pone.0315407)
Supplement: Attachment — Submitted filename: renamed_b2ae5.docx [file pone.0315407.s002.docx]

**Response to Reviewer´s comments**

**Journal requirements**

| No. | Comment | Response |
| --- | --- | --- |
| 1 | Please ensure that your manuscript meets PLOS ONE's style requirements, including those for file naming. The PLOS ONE style templates can be found at  https://journals.plos.org/plosone/s/file?id=wjVg/PLOSOne_formatting_sample_main_body.pdf and  https://journals.plos.org/plosone/s/file?id=ba62/PLOSOne_formatting_sample_title_authors_affiliations.pdf | Thank you. The authors confirm that the PLOS ONE´s style requirements are now met in the revised manuscript. |
| 2 | Please note that PLOS ONE has specific guidelines on code sharing for submissions in which author-generated code underpins the findings in the manuscript. In these cases, all author-generated code must be made available without restrictions upon publication of the work. Please review our guidelines at https://journals.plos.org/plosone/s/materials-and-software-sharing#loc-sharing-code and ensure that your code is shared in a way that follows best practice and facilitates reproducibility and reuse. | We have placed all the data on GitHub and now it is publically available. We have placed the information of the access in the manuscript. The data is available at: <https://github.com/tayyabawan786/covid19-project/tree/main> |
| 3 | Thank you for stating the following in the Acknowledgments Section of your manuscript:   "The authors would like to thanks the financial support from Tecnologico de Monterrey through the “Challenge-Based Research Funding Program 2022”. Project ID # I003 - IFE001 - C2-T3 – T"  We note that you have provided funding information that is not currently declared in your Funding Statement. However, funding information should not appear in the Acknowledgments section or other areas of your manuscript. We will only publish funding information present in the Funding Statement section of the online submission form.  Please remove any funding-related text from the manuscript and let us know how you would like to update your Funding Statement. Currently, your Funding Statement reads as follows:   "The author(s) received no specific funding for this work."  Please include your amended statements within your cover letter; we will change the online submission form on your behalf. | The authors disagree with this information. The financial support through these projects is only for the open-access publication fee, however, the research received no funding for the execution of the research. Therefore, it would be inappropriate to put the given statement within the funding section to not create confusion. Therefore, this funding is just placed in the Acknowledgement section. |
| 4 | In the online submission form, you indicated that "Data can be shared on request". All PLOS journals now require all data underlying the findings described in their manuscript to be freely available to other researchers, either 1. In a public repository, 2. Within the manuscript itself, or 3. Uploaded as supplementary information. This policy applies to all data except where public deposition would breach compliance with the protocol approved by your research ethics board. If your data cannot be made publicly available for ethical or legal reasons (e.g., public availability would compromise patient privacy), please explain your reasons on resubmission and your exemption request will be escalated for approval. | Thanks for your asking about dataset here is the link of dataset <https://github.com/tayyabawan786/covid19-project/tree/main/dataset>  The dataset is publicly available on our github repository |

**Reviewer # 1**

| No. | Comment | Response |
| --- | --- | --- |
| 1 | The abstracts introductory portion is too long. Readers expect to see more detail of the methodology, results, and conclusion in the abstract. The abstract need to be greatly improved. The abstract does not show that the authors achieved much as there is no numerical justification to back the author’s claims or results of comparative analysis to show superior performance. | Thank you for your kind recommendation to improve the abstract section. The authors confirm that more details are now added for the methodology, results, and conclusion in the abstract. Apart, numerical justification to back our claims are also added. |
| 2 | In the introduction, the authors should explain why they did it (motivation) discussing the possible outcome. Readers are primarily interested in the motivation and outcome of your research. Therefore, a good introduction should contain: a. What is the problem to be solved? b. Are there any existing solutions? c. Which is the best? d. What is the main limitation of the best and existing approaches? e. What do you hope to change or propose to make it better? f. How is the paper structured? | Thank you for presenting the information in a simplified format. Here is the stepwise answer to your recommendations for a good introduction.   a. The problem which is addressed to be solved is further explained in the second paragraph of the Introduction section. b. The existing solutions are explained in section 2: literature review and related works. c. Table 1 summarizes the comparison between different approaches to identify the best method. d. The main limitation of the best and the existing approaches is to not be using an extensive list of algorithms like identified in the last row of Table 1. e. We propose to use a set of machine learning and deep learning methods to improve the accuracy as identified in the last row of Table 1. f. The structure of the paper is now added in the last paragraph of section 2.1. |
| 3 | Please clearly highlight how your work advances the field from the present state of knowledge and you should provide a clear justification for your work which should be stated at the end of literature review/ related works. The impact or advancement of the work can also appear in the conclusion. | Thank you for your concern. Table 1 outlines the differences in the present state of knowledge, and we have added another section 2.1 which is exclusively dedicated for the research contributions outlined in the literature review. |
| 4 | Authors should use a flowchart to illustrate the Procedure to Produce the FLDI index. This will help readers to understand the procedure better. | Thank you for the suggestion. Figure 1 presents an integral methodological diagram which can help the readers to understand the procedure better. |
| 5 | Related works section is not sufficient. The authors should improve on this section as they have left many papers out. Normally, it’s the gaps in work of others that the authors are expected to fill. Therefore, at the end of your review section state the problems in this field with appropriate reference and tell readers which one your work addresses. The authors should consult and cite: (i) Detection and Classification of COVID-19 Disease from X-ray Images Using Convolutional Neural Networks and Histogram of Oriented Gradients. Biomedical Signal Processing and Control, 103530, Vol. 74, pp. 1-11, 2022. DOI: 10.1016/j.bspc.2022.103530 (ii) Prediction of COVID-19 Outbreak with Current Substantiation Using Machine Learning Algorithms. Intelligent Interactive Multimedia Systems for e-Healthcare Applications. Springer, Singapore, 2022. https://doi.org/10.1007/978-981-16-6542-4_10 (iii) CIA-CVD: cloud based image analysis for COVID-19 vaccination distribution. Journal of Cloud Computing. Vol. 12, 163. DOI: 10.1186/s13677-023-00539-y (iv) PETLFC: Parallel ensemble transfer learning based framework for COVID-19 differentiation and prediction using deep convolutional neural network models. Multimedia Tools and Applications. DOI: 10.1007/s11042-023-16084-4 (v) X-Ray image-based COVID-19 detection using deep learning. Multimedia Tools and Applications, Vol. 82, pp. 44507–44525. DOI: 10.1007/s11042-023-15389-8 (vi) Detection of Corona Virus Disease Using a Novel Machine Learning Approach. 2021 International Conference on Decision Aid Sciences and Application (DASA), pp. 587-590, 2021. DOI: 10.1109/DASA53625.2021.9682267. | The authors agree with the feedback of the kind reviewer. The authors have significantly improved the related work section. The mentioned articles are helpful for the authors to identify the gaps in the work of other researchers which are eventually cited just before Table 1. Thank you. |
| 6 | Most of the figures in this paper are not clear enough. The authors should endeavour to change them. E.g. Figs. 1-4. | The authors confirm that all the figures from 1 to 4 have high quality. It is possible that the generated PDF by the PLOS ONE system is converting the images into a low-quality to save the memory space. |
| 7 | The authors need to discuss the results in Tables 1 and 2 better. The reason why the proposed technique performs better has not been explained. | Thanks for your highlighting this issue now table 1 and Table 2 explained and better technique also explained |
| 8 | There is no comparison of results with the existing works in this paper. This should be added for readers to see how your proposed method performs relative to other works. | The comparison of our experiments with state-of-the-art methods has been done in section comparison |
| 9 | It would be good for the authors to discuss other methods that different authors have used to take fake news. Authors should read and cite: - Detection of fake news and hate speech for Ethiopian languages: a systematic review of the approaches. Journal of Big Data, pp. 1-17. DOI: 10.1186/s40537-022-00619-x | Agreed. The authors have now placed this article into the related works section, just before Table 1. |
| 10 | The authors should structure the paper into abstract, introduction, literature review/related works, methodology, results and discussion, and conclusion. | Thank you for outlining the structure. The authors confirm that the paper is now structured into: abstract, introduction, literature review/related works, methodology, results and discussion, and conclusion and recommendation. |
| 11 | I was hoping to see more results and discussion as more results could be presented to make the work much appreciable. The authors are encouraged to reduce the plagiarism of the paper. | Thanks for your recommendation the results and discussion is completed and plagiarism is reduced |
| 12 | The Limitations of the proposed study need to be discussed before conclusion. | Thank you for your comment. The authors have now added the section of limitations of the proposed work as a section 5. |
| 13 | Some of the challenges encountered during the course of the study can be highlighted and future recommendations can be added at the end of the conclusion. Retitle conclusion as conclusion and recommendation. | Thank you for your recommendation the author added the future recommendation at the end of conclusion |
| 14 | The results and discussion section is very weak. The authors should endeavor to improve on this section. In the section of selection of local minima, what criteria did the authors use. Also what priors did the authors consider? What is the minimum and the maximum values? If these are suitable, do they work for different types of images or just the images under consideration? | Thanks we have improved the result and discussion and also added the section comparison and discuss our methodology has better results |
| 15 | Lastly, no comparison of results was presented with other state-of-the-art methods which have used machine learning techniques | The section comparison with previous studies is explained in details in new manuscript |

**Reviewer # 2**

Overall this is very interesting work in ML and DL based on Sentiment analysis, but the overall presentation of the work needs a major improvement. The current manuscript needs significant improvements. Some of the improvements need to be done in this manuscript are as follows:

| No. | Comment | Response |
| --- | --- | --- |
| 1 | The Motivation and Contribution Subsection needs to be added in the Introduction Section. The major contribution and novelty of the work need to be briefly discussed. | Thank you for your comment. The authors have now added the section of motivation and research contribution as a sub-section of section 2. |
| 2 | A separate Section Literature Review needs to be added and a recent state-of-the-art scheme needs to be discussed. | Agreed. A separate section with the name: Literature Review and related works is now added to the manuscript. |
| 3 | Various ML and DL-based approaches and techniques need to be discussed in in New Section Background Detail. | Agreed. The authors have now added a new section 1.1. Background in the manuscript where we discussed sentiment analysis along with various ML and DL based approaches. |
| 4 | Data Labeling and vectorization steps are considered in this work or not? Proper discussion is needed, as these are the major steps in sentiment analysis. For reference, the author may refer to the following work:Gupta, P., Kumar, S., Suman, R. R., & Kumar, V. (2020). Sentiment analysis of lockdown in india during covid-19: A case study on twitter. IEEE Transactions on Computational Social Systems, 8(4), 992-1002.  Jayasurya, G. G., Kumar, S., Singh, B. K., & Kumar, V. (2021). Analysis of public sentiment on COVID-19 vaccination using twitter. IEEE Transactions on Computational Social Systems, 9(4), 1101-1111. | Thanks for your highlighting this issue we use TFIDF for text vectorization and dataset is is already labeled by annotators when its obtained form the the recourse.  The references mentioned are now cited in the research article which were very helpful to understand the major steps in sentiment analysis. |
| 5 | In section IV the proper equation is needed for accuracy, recall, precision, and F1 score. Every variable used in the equation needs proper discussion. | Thank you for your feedback. The authors have updated the proper equations accuracy, recall, precision, and F1 score. |
| 6 | In Table 3, what is the random state for the ML techniques? All these values change with the random state. It is recommended to check the result with varying random states or perform k-fold cross-validation. | Thanks for your inquiry In machine learning, "random state" controls random initialization to maintain consistency in results and ensures repeatability across runs for a consistent assessment of model performance. We use random_state=42 that provides repeatable outcomes by starting with a fixed random seed. |
| 7 | Further the result of this work needs to be compared with some of the recent state-of-the-art work. | The section comparison with previous studies is explained in details in new manuscript |
| 8 | What is the novelty of this work? There are several works in which this type of work is reported. So, it is recommended to add the novelty of the work in the motivation and contribution section. | Agreed. Thank you. We have added a dedicated section 2.1. related to the motivation and research contribution. |
