# Supplementary material for: Machine and deep learning algorithms for sentiment analysis during COVID-19: A vision to create fake news resistant society
Source: PLoS One. 2024 Dec 19;19(12):e0315407. doi: 10.1371/journal.pone.0315407 (PMC11658524; doi:10.1371/journal.pone.0315407)
Supplement: Attachment — Submitted filename: renamed_5ce28.docx [file pone.0315407.s003.docx]

**Response to Reviewer´s comments**

**Journal requirements**

| No. | Comment | Response |
| --- | --- | --- |
| 1 | Please review your reference list to ensure that it is complete and correct. If you have cited papers that have been retracted, please include the rationale for doing so in the manuscript text, or remove these references and replace them with relevant current references. Any changes to the reference list should be mentioned in the rebuttal letter that accompanies your revised manuscript. If you need to cite a retracted article, indicate the article’s retracted status in the References list and also include a citation and full reference for the retraction notice. | Thank you for your valuable feedback regarding the reference list of our manuscript. We appreciate your thorough review and have carefully examined all references to ensure their completeness and accuracy. Upon reviewing the reference list, we can confirm that all citations are correct and accurately represent the sources cited in the manuscript. We have taken great care to verify each reference against its original source to maintain the integrity of our work. |

**Reviewer # 3**

| No. | Comment | Response |
| --- | --- | --- |
| 1 | I appreciated the efforts made by the authors to compare the methods in sentiment analysis related to supervised ML and DL. Since this is the revised version, it is not fair to further point out that the comparison between SVM/RF etc. vs. CNN, LSTM, GRU addresses the state-of-the-art technique like LLMs. A series of recent research shows that some of these fine-tuned LLMs or even zero-short learning using GPT outperform traditional methods. This paper should have improved greatly if they can further include these methods. | Thank you for sharing your concerns while recognizing that the manuscript has already undergone revisions. The authors argue that incorporating new methodologies at this stage would fundamentally alter the manuscript's narrative, structure, and possibly its conclusions. Such changes would essentially revert the manuscript to its initial draft form. In light of the manuscript's current scope, the authors have chosen to proceed with the existing supervised Machine Learning (ML) and Deep Learning (DL) methods. Nevertheless, we have now explicitly mentioned in the section on future research that exploring advanced Transformer-based architectures and larger language models, such as those in the GPT (Generative Pre-trained Transformer) series, including GPT, T5, and other Large Language Models (LLMs), offers significant potential for a more profound analysis of complex linguistic patterns, especially in studies examining COVID-19 misinformation. |

**Reviewer # 4**

This paper introduces a technique for detecting misinformation related to the COVID-19 pandemic. The experimental results indicate that the BiGRU deep learning classifier demonstrates a higher accuracy and efficiency than six machine learning and two deep learning classifiers. In general, the paper is well written and easy to understand. The reviewer believe that this paper can be accepted for publication, however, I do have some comments, in particular:

| No. | Comment | Response |
| --- | --- | --- |
| 1 | Some of key publications in this field are missing in the introduction section. It is suggested to add them in. | Thank you for your review regarding the key publications in the introduction section of our manuscript. We appreciate your suggestion and have thoroughly reviewed the existing references to ensure their relevance and accuracy in representing the field. The introduction section combined with the background section, and literature review summarizes the work of more than 50 research articles in this area. We have ensured that all referenced publications are correct and align with the scope and focus of our research. However, if you have specific key publications in mind that you believe should be included, please feel free to provide us with their details. We would be happy to consider incorporating them into the introduction section, if appropriate. |
| 2 | Some references are not formatted correctly. | Thank you for your concern. We have revised the format of the references which apparently seems to have all the useful information, eventually, the production department of the journal can have a better look into it. |
