# Supplementary material for: Machine and deep learning algorithms for sentiment analysis during COVID-19: A vision to create fake news resistant society
Source: PLoS One. 2024 Dec 19;19(12):e0315407. doi: 10.1371/journal.pone.0315407 (PMC11658524; doi:10.1371/journal.pone.0315407)
Supplement: Attachment — Submitted filename: Response to Reviewer.docx [file pone.0315407.s004.docx]

**Response to Reviewer´s comments**

**Reviewer no. 3**

| No. | Comment | Response |
| --- | --- | --- |
| 1 | Thanks for adding the potential usage of generative AI tools such as GPT models at the end of discussion. Again, when dealing with fake news detection, the state-of-the-art technique of deep learning might be constantly changing. The author(s) need to acknowledge how other scholars have used LLMs (e.g., BERT model, Roberta, longormer, etc.) in the field for fake news detection (which is absent in the current version). | Thank you very much for this important point we have added a new subsection Recent Advances with Large Language Models in this section we have described this in detail and acknowledged the recent work of other scholars with Transformers and LLMs. |

**Reviewer no. 5**

| No. | Comment | Response |
| --- | --- | --- |
| 1 | Abstract: Can the authors begin by briefly introducing the significant problem of fake news to emphasize the impact of this study since the beginning? | Thank you for the observation. The abstract has a context of problem formulation and then it leads towards the significant problem of fake news to explain the impact of this study. |
| 2 | Table 1: Table 1 will be very useful for future researchers. However, the authors should consider the consistency of the summarized data in the table. Some cells provided very detailed details, while others showed only the techniques' names. | Thanks for highlighting the important task. Now we have summarized the content and bought consistency in table almost now every cell has balanced detail. |
| 3 | Data set: The retrieving date of the data should be mentioned. | Thanks for asking for the retrieving date for the dataset which we obtained on 28-08-2023 for our work. The details are now mentioned in section 2.1. |
| 4 | Data set: Some explanation of the data set should be added. For example, when and how Kaggle collected them. | Thanks for your kind question, the dataset was obtained from news articles by scraping them from different websites and annotation was done manually by the experts. The dataset was originally published by Dr Malar Kodi (Link: <https://www.kaggle.com/datasets/csmalarkodi/covid-fake-news-dataset>) |
| 5 | Since there is a keyword "Computational Thinking," this term should be used or mentioned in the contents. | Thank you. The keyword is now removed. |
