# Supplementary material for: Machine and deep learning algorithms for sentiment analysis during COVID-19: A vision to create fake news resistant society
Source: PLoS One. 2024 Dec 19;19(12):e0315407. doi: 10.1371/journal.pone.0315407 (PMC11658524; doi:10.1371/journal.pone.0315407)
Supplement: Attachment — Submitted filename: Response to Reviewer.docx [file pone.0315407.s005.docx]

**Response to Reviewer´s comments**

**Editor comment:**

| No. | Comment | Response |
| --- | --- | --- |
| 1 | Please review your reference list to ensure that it is complete and correct. If you have cited papers that have been retracted, please include the rationale for doing so in the manuscript text, or remove these references and replace them with relevant current references. Any changes to the reference list should be mentioned in the rebuttal letter that accompanies your revised manuscript. If you need to cite a retracted article, indicate the article’s retracted status in the References list and also include a citation and full reference for the retraction notice. | Thank you for your valuable feedback. We confirm that all the references fulfill the requirement as proposed by the editor. |

**Reviewer no. 5**

| No. | Comment | Response |
| --- | --- | --- |
| 1 | All the changes suggested by the reviwers are positively addredesed by thr authors. But, I will suggest authors to proof read the manuscript carefully.  For example: " 2.41. Support Vector Machines ". It will be 2.4.1. Similarrly Proper abbrebration is required in manuscript. For example : iN section 2.4.1 abbrebration of SVM is given thrice. | Thank you for the helpful suggestions. We have carefully proofread the manuscript, making all necessary formatting corrections, such as revising section numbers (e.g., correcting "2.41" to "2.4.1") and ensuring consistent and appropriate use of abbreviations like "SVM" throughout the document. All changes were made according to your recommendations, and the manuscript is now formatted as per the guidelines. |
